# Construction of a Novel circRNA/miRNA/mRNA Regulatory Network to Explore the Potential Pathogenesis of Wilson’s Disease

**DOI:** 10.3389/fphar.2022.905513

**Published:** 2022-06-15

**Authors:** Taohua Wei, Nannan Qian, Wenming Yang, Yue Yang, Jie Liu, Wenjie Hao, Ting Cheng, Ran Yang, Wei Dong, Yulong Yang

**Affiliations:** ^1^ Department of Neurology, The First Affiliated Hospital of Anhui University of Chinese Medicine, Hefei, China; ^2^ Key Laboratory of Xin’an Medicine of the Ministry of Education, Anhui University of Chinese Medicine, Hefei, China; ^3^ Graduate School, Anhui University of Chinese Medicine, Hefei, China; ^4^ Institute for Medical Virology, Goethe University Frankfurt am Main, Frankfurt, Germany; ^5^ Graduate School, Guangzhou University of Chinese Medicine, Guangzhou, China; ^6^ Department of Oncology, Affiliated Hospital of Nanjing University of Chinese Medicine, Nanjing, China

**Keywords:** Wilson’s disease, regulatory network, circRNA, miRNA, mRNA, biomarkers

## Abstract

Studies show that non-coding RNAs, especially microRNAs (miRNAs) and circular RNAs (circRNAs), and protein-coding genes are involved in the pathophysiology of multi-organ damage caused by Wilson’s disease (WD). However, circRNA expression profiles and their role in initiation and progression of WD kidney injury remain largely unclear at present. Here, we explored potential critical protein-coding genes, miRNAs, and circRNAs, as well as identify competitive endogenous RNAs (ceRNAs) in a WD mouse model by high-throughput sequencing. We investigated the expression profiles of circRNAs, miRNAs, and protein-coding genes, and identified 32 DEcircRs, 45 DEmiRs, and 1623 DEPs. Identified DEcircRs, DEmiRs, and DEPs were used to construct a ceRNA network, which consisted of 15 DEcircRNAs (four upregulated and 11 downregulated), 18 DEmiRNAs (14 upregulated and four downregulated), and 352 DEmRNAs (205 upregulated and 147 downregulated). Further experiments proved that mmu_circ_0001333 and mmu_circ_0000355 acted as sponges of miR-92b-5p, miR-107-3p, and miR-187-3p to regulate the expression of genes including *Smad9*, *Mapk10*, and *Aldh3a2*, which may participate in WD-related kidney injury. Taken together, this study identified the circRNA/miRNA/mRNA network involved in kidney failure in WD, which may serve as a potential biomarker for the pathogenesis of WD.

## Introduction

Wilson’s disease (WD), first described in 1912 by Wilson (1912), is a rare autosomal recessive disease associated with copper metabolism. *ATP7B*, encoding P1B-type copper transport adenosine triphosphatase (ATPase), is mutated in WD (Bull et al., 1993; Tanzi et al., 1993). ATP7B is expressed in many organs including the liver, brain, kidney, placenta, breast, and testes, and it is also involved in tissues damage in these organs (Bull et al., 1993; Tanzi et al., 1993). The clinical manifestations of the disease mainly include liver and kidney damage, extrapyramidal symptoms, and corneal pigmentation. It was previously shown that the accumulation of copper in the kidney of Long Evans Cinnamon (LEC) rats was 10–20 times that of the normal control group after 12–18 weeks (Hayashi et al., 2006). Although liver and brain damage are the most common and typical ones, studies have shown that kidney damage occurs earlier, especially with the increase of copper concentration, due to single-strand DNA breaks in the renal cortex (Hayashi et al., 2005; Hayashi et al., 2006).

Increasing evidence shows that non-coding RNAs, especially microRNAs (miRNAs), long non-coding RNAs (lncRNAs), and circular RNAs (circRNAs), are involved in the pathophysiology of almost all organs, including liver damage caused by WD (Zhang et al., 2021). CircRNAs are evolutionarily conserved transcripts that are characterized by covalently linked 5′ and 3′ ends, which are derived from mRNA pre-splicing (Chen, 2016). Therefore, their life span is usually extended continuously from several hours to 6 days, making them more stable than linear-coding or non-coding mRNAs (Memczak et al., 2013) circRNAs may affect their canonical splicing because of back-splicing (Chen, 2020), and also regulate gene expression in the nucleus, acting as bait for miRNAs and proteins, and as a scaffold for circRNA-protein complexes. Some circRNAs may be used as translation templates or sources of pseudogenes. Interestingly, a circRNA was previously shown to compete with proteins for DNA binding. Recent studies have shown that circRNAs act as competitive endogenous RNAs (ceRNAs) by sponging microRNAs (miRNAs) through multiple miRNA binding sites, and inhibit miRNAs, regulating their downstream targets. To date, many circRNAs have been proven to affect tumor progression (Abdollahzadeh et al., 2019; Liang et al., 2020). Interestingly, some studies have also reported this mechanism in kidney diseases (Jin et al., 2020). For example, circTLK1 promotes the proliferation and metastasis of renal cell carcinoma by sponging miR-136-5p (Li et al., 2020). Shi et al. (2020) reported that circVMA21 ameliorates sepsis-associated acute kidney injury by regulating the miR-9-3p/SMG1/inflammation axis and oxidative stress. CircRNA_010383 acts as a sponge for miR-135a and its downregulation contributes to renal fibrosis in diabetic nephropathy (Peng et al., 2021). However, so far, the roles and mechanisms of circRNAs in WD, especially in WD-related kidney damage, has not been elucidated.

Therefore, in this study, after identifying the profiles of circRNAs, miRNAs, and mRNAs, we performed a comprehensive analysis of their roles in WD. We also constructed a ceRNA regulatory network. Several different analyses, not just functional enrichment and protein-protein interaction (PPI) analyses, were performed. Furthermore, the validation for some differentially expressed protein-coding genes (DEPs), miRNAs (DEmiRs), and circRNAs (DEcircRs), which are associated with the ceRNA network, were identified *via* quantitative real-time polymerase chain reaction (qRT-PCR).

## Materials and Methods

### Animal Experiments

Male Toxic Milk (TX-J) mice were obtained from the Jackson Laboratory (United States). The genotype was confirmed *via* Sanger sequencing (Wei et al., 2021). The TX-J model was prepared as described previously (Wei et al., 2021) and the procedure conformed with the National Institutes of Health Animal Care and Welfare Guidelines. All mice were sacrificed *via* intraperitoneal injection of sodium pentobarbital. The experimental procedure was approved by the Animal Experiment Scientific Research Committee of Anhui University of Traditional Chinese Medicine (AHUCM-mouse-2020027).

### Haematoxylin and Eosin and Dithiooxamide Staining

After the animals were sacrificed, kidney tissues were collected and fixed in 4% formaldehyde solution for 48 h. Kidney tissues were dehydrated with different concentrations of xylene and embedded in paraffin. They were then stained with HE. Moreover, kidney tissue sections were stained with 0.1% dithiooxamide ethanol solution in a 37°C water bath for 3 days. Then, a light microscope (Olympus, Japan) was used for visualizing the sections.

### High-Throughput RNA Sequencing of circRNAs, miRNAs, and mRNAs in Wilson’s Disease-Related Kidney Damage

Total RNA was extracted from mouse kidney tissues using MiRNeasy Mini Kit (Cat#217004, Qiagen, Germany). Then, RNA was purified using VAHTS RNA Clean Beads (N412-01, Vazyme, CN), DNase I, and RNase-free (EN401, Vazyme, CN). Quality control (QC) was achieved using NanoDrop 2100 (Thermo Fisher Scientific, United States) and Agilent Bioanalyzer 4200 (Agilent Technologies, United States).

Small RNA (sRNA) (∼21 nucleotides) and whole transcriptome libraries were generated using the QIAseq miRNA Library Kit (Cat#331505, Qiagen, Germany) and VAHTS Total RNA-seq (H/M/R) Library Prep Kit (NR603-01, Vazyme, China) following the manufacturers’ instructions. All libraries were quantified using Agilent 2100 Bioanalyzer and Qubit^®^ 3.0 fluorometer (Invitrogen; Thermo Fisher Scientific, Inc.). sRNA libraries and whole transcriptome libraries were sequenced on the Illumina Xten and Illumina NovaSeq 6000 (Illumina Inc., San Diego, CA, United States), respectively.

### Quantitative and Differential Analyses of DEcircR, DEmiRs, and Differentially Expressed Protein-Coding Genes

FastQC (v. 0.11.3) (Andrews, 2010) was employed to achieve QC of the raw RNA-seq reads. Trimming was performed using seqtk2 (H. Li, https://github.com/lh3/seqtk/) to remove the known Illumina TruSeq adapter sequences, poor reads, and ribosomal RNA reads. Meanwhile, mapping was also performed using the BWA-MEM software (version: 2.0.4) with trimmed reads. CIRI software (Gao et al., 2015) was used to predict circRNAs. Known circRNAs were identified based on the matching location using circBase (Glažar et al., 2014), and counts were normalized using SRPBM (Li et al., 2015). For sRNA-seq data, raw reads were qualified and filtered using the FASTX software (version 0.0.13) (Hannon, 2010). Then, clean reads were mapped to the mouse genome (mm10) (Flicek et al., 2014) and miRBase (version 21) (Kozomara and Griffiths-Jones, 2014) was used for miRNA annotation *via* Bowtie (Langmead et al., 2009). miRNA counts were normalized to transcripts per million reads (Robinson et al., 2010; Nikolayeva and Robinson, 2014). EdgeR (Robinson et al., 2010) was used to identify differentially expressed RNAs (DERNAs) including circRNAs, miRNAs, and protein-coding genes using the high-throughput sequencing data. A |log2FoldChange| of > 1 and a *p*-value of < 0.05 were considered significant. Volcano maps of DEcircRs, DEmiRs, and DEPs were constructed using ggplot2 (Ginestet, 2011). A hierarchical two-way cluster analysis was performed to compare the clusters, and heatmaps were drawn using the pheatmap package (Kolde, 2015).

### Target Prediction and Construction of the ceRNA Network

DEmiR sequences were obtained from the miRbase (Kozomara and Griffiths-Jones, 2014). Sequences of known DEcircRs were obtained from circBase (Glažar et al., 2014), and novel DEcircRs were obtained from ensembl based on chromosome location (Flicek et al., 2014). Prediction of miRNAs targeted by DEcircRs was performed using the software based on miRanda (Betel et al., 2008) and RNAhybrid (Rehmsmeier et al., 2004), and DEcircR-miRNA pairs were arranged to construct the circRNA-miRNA network. Meanwhile, the correlation between DEcircRs and DEmiRs were calculated based on the normalized expression of DEcircRs and DEmiRs, and the ones with a negative correlation were retained. DEmiR protein-coding targets were obtained from the mirTarbase (Chou et al., 2018). Similarly, the correlation between DEmiRs and DEPs was calculated based on the normalized expression of DEmiRs and DEPs and the negative correlation were retained to construct the miRNA-mRNA network. Finally, the circRNA-miRNA-network was visualized using Cytoscape (Shannon et al., 2003).

### Functional and Pathway Enrichment Analysis

Gene Ontology (GO) at three levels biological process (BP), cellular component (CC) and molecular function (MF) (Consortium, 2004)) and Kyoto Encyclopedia of Genes and Genomes (KEGG) enrichment analyses (Kanehisa and Goto, 2000) were performed using clusterProfiler (Yu et al., 2012). The DEPs and the targets of the ceRNA network were used for the functional enrichment analyses. The terms, with a *p*-value of < 0.05, were considered statistically significant.

### Protein-Protein Interaction Analysis

The interactions among DEPs or the targets of the ceRNA network were identified using the Search Tool for the Retrieval of Interacting Genes/Proteins (STRING) (Szklarczyk et al., 2017) with the default minimum required interaction score (≥0.4), and the PPI network was constructed by Cytoscape (Shannon et al., 2003).

### Validation of DEcircRs, DEmiRs, and Differentially Expressed Protein-Coding Genes *Via* qRT-PCR

After total RNA was extracted from kidney tissues, cDNA was generated *via* reverse transcription. According to the results of the functional enrichment, PPI analysis, and the ceRNA network, we chose two circRNAs, three miRNAs, and three protein-coding genes for qRT-PCR verification using 2X Universal SYBR Green Fast qPCR Mix (RK21203, Abclonal, CN) with specific primers on six kidney tissues from TX-J mice (Model group) and control mice (CN group) were used to confirm the circRNA ceRNA network. Relative expression levels were calculated using the 2^−ΔΔCt^ method (Livak and Schmittgen, 2001).

### Statistical Analysis

qRT-PCR results were analyzed using GraphPad software *via* the Student’s *t*-test, with *p* < 0.05 accepted as statistically significant. The data were shown as the mean ± standard error of the mean.

## Results

### Construction of the Mouse Model

**FIGURE 1:**
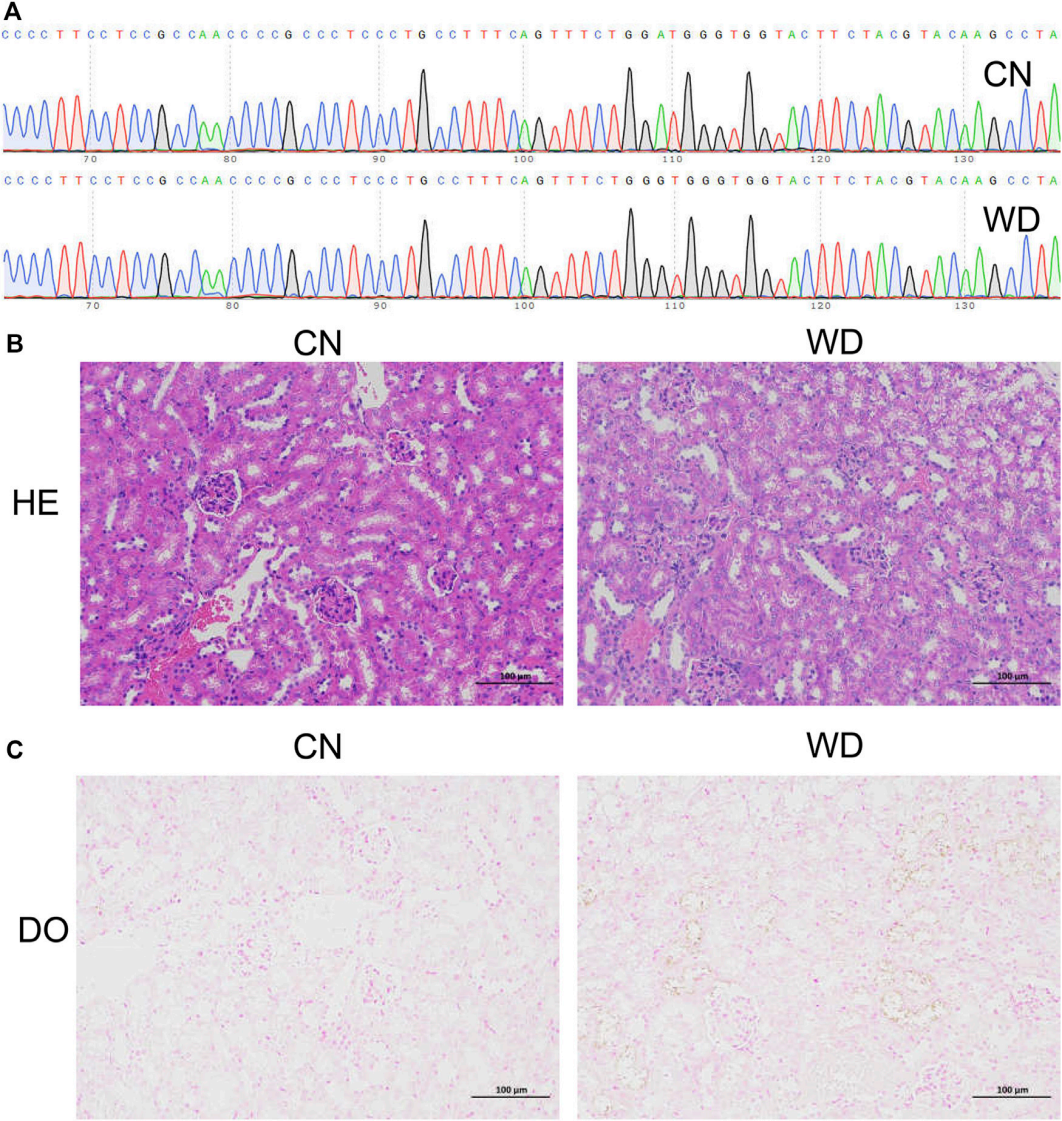
Confirmation of the WD TX-J mouse model with kidney injury. **(A)** Sanger sequencing of the TX-J model mice (diploid mutation type, Model group) and control mice (diploid wild type, CN group). **(B)** HE and **(C)** DO pathological staining of kidneys in Model and CN groups. HE, Haematoxylin and eosin; DO, Dithio oxamide.

Sanger sequencing was used to detect the genotypes of all mice, and diploid mutant and wild type mice were identified as TX-J (Model group) and control mice (CN group), respectively (Figure 1A). The results of HE staining (Figure 1B) showed that the kidneys were normal in the CN group, whereas tubular cell necrosis, cytoplasmic vacuoles, loss of brush border, and tubular dilation were observed in WD mice. Copper-containing granules were distinctly black after tissue sections were incubated for 72 h, and we observed more granules in the kidneys of WD than in those of the CN group (Figure 1C). This proved that the TX-J mouse kidney was injured and copper was present.

### Identification of Differentially Expressed Protein-Coding Genes and Functional Enrichment Analysis in the Kidneys of Wilson’s Disease Mice

**FIGURE 2:**
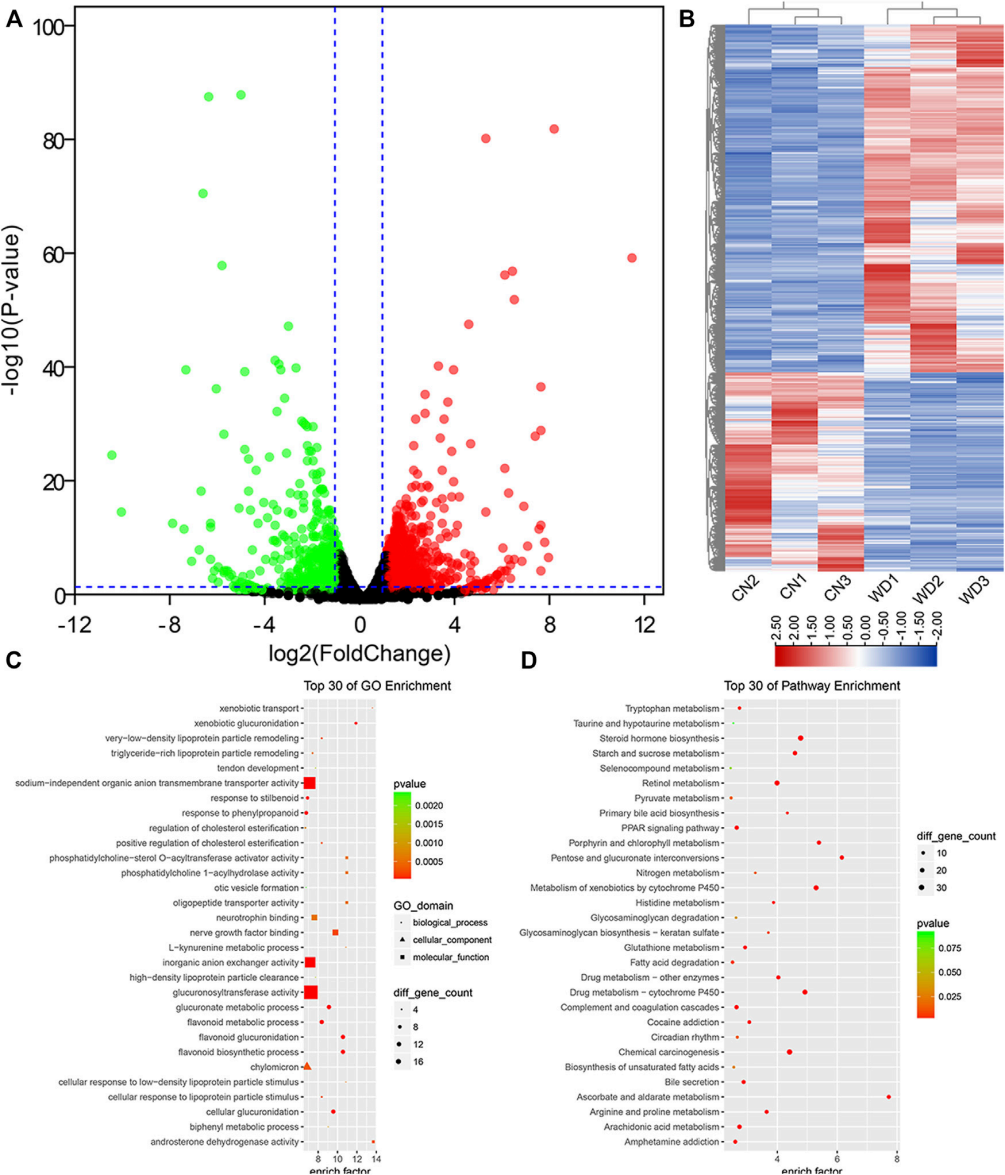
DEPs between the WD and control groups in the kidneys and functional enrichment analysis. **(A)** DEPs displayed on a volcano plot. Blue and red indicate > two folds decrease or increase in expression, respectively (*p* < 0.05). Gray indicates no significant difference. **(B)** Hierarchical clustering; numbers indicate the samples used for RNA-seq. **(C)** Top 30 GO enrichment terms of DEPs. **(D)** Top 30 KEGG pathway enrichment terms of DEPs. DEPs, differentially expressed protein-coding genes.

In total, we identified 22,430 protein-coding genes from six samples. Further, 1,623 DEMs were filtered from the kidneys of WD mice, including 1,034 upregulated and 589 downregulated DEMs (*p* < 0.05, log_2_|fold change| > 1). The results were visualized *via* volcano and hierarchical clustering plots (Figures 2A,B).

To further identify the 1,623 DEPs, GO enrichment analysis was performed, which revealed that 838 GO terms, were significantly (*p* < 0.05) enriched, including 594 BP terms, 76 CC terms, and 168 MF terms. The top ten enriched BP terms were organic anion transport (GO: 0015711), flavonoid biosynthetic process (GO: 0009813), flavonoid glucuronidation (GO: 0052696), cellular glucuronidation (GO: 0052695), anion transport (GO: 0006820), ion transport (GO: 0006811), glucuronate metabolic process (GO: 0019585), flavonoid metabolic process (GO: 0009812), transmembrane transport (GO: 0055085), and monocarboxylic acid metabolic process (GO: 0032787). Extracellular space (GO: 0005615), extracellular region (GO: 0005576), basolateral plasma membrane (GO: 0016323), intrinsic component of plasma membrane (GO: 0031226), extracellular matrix (GO: 0031012), integral component of plasma membrane (GO: 0005887), cell surface (GO: 0009986), anchored component of membrane (GO: 0031225), axon (GO: 0030424), and ion channel complex (GO: 0034702) were the top ten enriched CC terms. The top ten enriched MF terms were secondary active transmembrane transporter activity (GO: 0015291), transmembrane transporter activity (GO: 0022857), transporter activity (GO: 0005215), substrate-specific transmembrane transporter activity (GO: 0022891), ion transmembrane transporter activity (GO: 0015075), glucuronosyltransferase activity (GO: 0015020), organic anion transmembrane transporter activity (GO: 0008514), anion transmembrane transporter activity (GO: 0008509), sodium-independent organic anion transmembrane transporter activity (GO: 0015347), and oxidoreductase activity, acting on CH-OH group of donors (GO: 0016614). According to the enrichment factor, we showed the top 30 DEG-related GO terms in Figure 2C.

To interpret gene functions and interactions, KEGG pathway analysis was performed using clusterProfiler with 1,623 differentially expressed genes (DEGs); 264 pathway terms were gained, particularly 43 terms with *p* < 0.05. We found that many metabolic pathways were enriched, such as steroid hormone, ascorbate and aldarate, retinol, fatty acid, porphyrin and chlorophyll, starch and sucrose, arginine and proline, arachidonic acid, glutathione, and histidine. In addition, we also identified that many other important pathways were significantly enriched, including complement and coagulation cascades, PPAR signaling pathway, cAMP signaling pathway, circadian entrainment, ECM-receptor interaction, MAPK signaling pathway, TGF-beta signaling pathway, inflammatory mediator regulation of TRP channels, ABC transporters, circadian rhythm, and signaling pathways regulating pluripotency of stem cells. According to the enrichment factor, we showed the top 30 downregulated KEGG pathway terms in Figure 2D.

**FIGURE 3:**
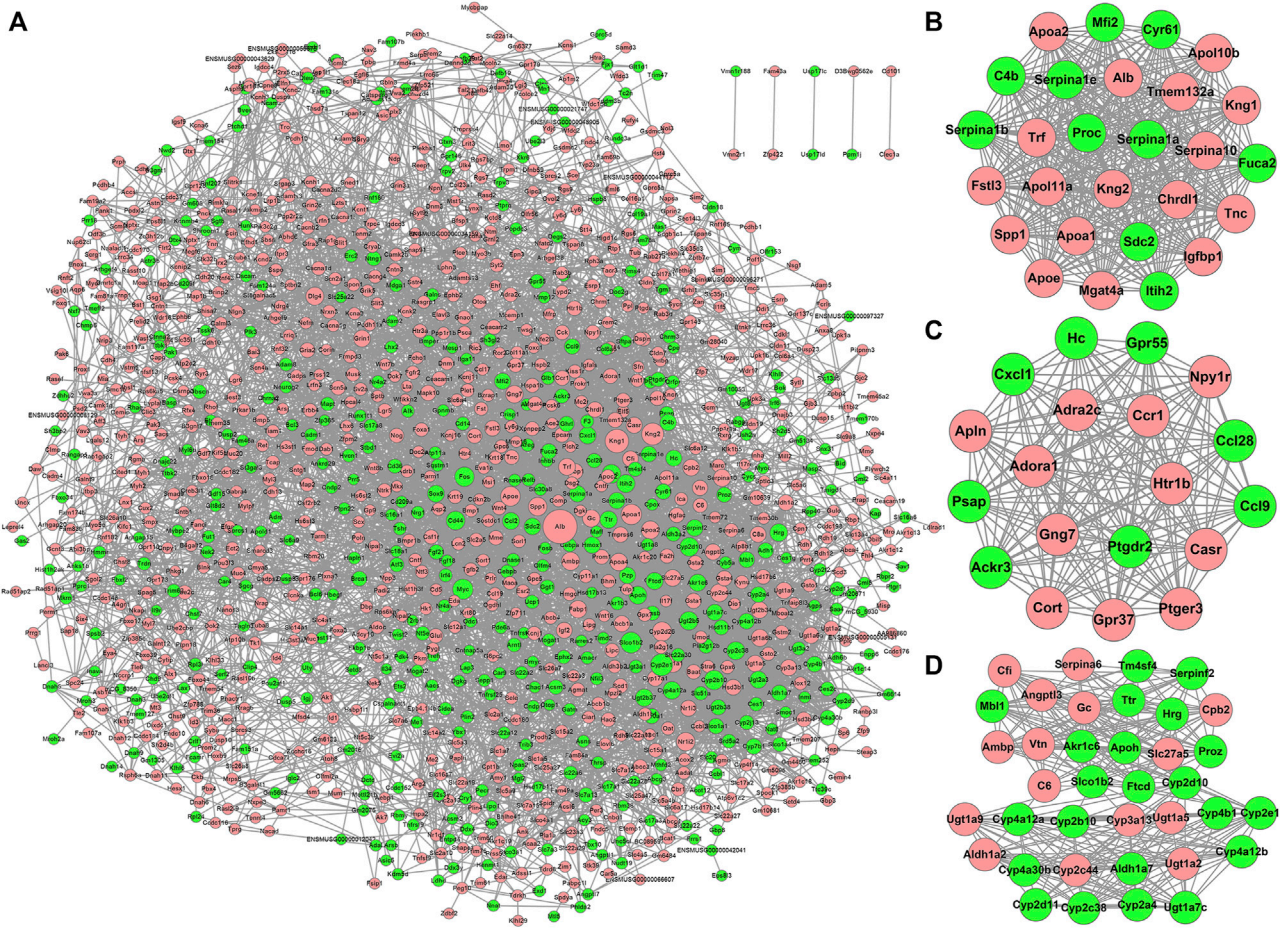
PPI network of DEPs and three key network modules **(A–D)** for DEPs in WD kidneys. Node size is related to the node degree. Pink and green nodes denote upregulated and downregulated protein-coding genes, respectively. PPI, protein-protein interaction; DEPs, differentially expressed protein-coding genes.

To understand the interactions between proteins translated from mRNAs, the PPI network of DEGs was constructed according to the information on the STRING database. The PPI network consisted of 1,337 DEGs and 6,840 pairs of interactions (Figure 3A). Dozens of gene nodes showed high connectivity (degree >50), such as albumin (*Alb,* degree = 163), kininogen 1 (*Kng1*, degree = 89), cytochrome P450, family 2, subfamily e, polypeptide 1 (*Cyp2e1*, degree = 79), kininogen 2 (*Kng2*, degree = 78), solute carrier organic anion transporter family, member 1b2 (*Slco1b2*, degree = 71), apolipoprotein A-I (*Apoa1*, degree = 68), myelocytomatosis oncogene (*Myc*, degree = 67), apolipoprotein E (*Apoe*, degree = 62), cytochrome P450, family 2, subfamily b, polypeptide 10 (*Cyp2b10*, degree = 61), and FBJ osteosarcoma oncogene (*Fos*, degree = 61).

Using the MCODE plugin with default criteria, ten modules were identified. The details of all modules were sorted in descending order of the module score. Based on the module scores, the top three modules, modules one to three, were picked for the visualization of the module network (Figures 3B–D).

### Identification of DEmiRs and the Construction of the miRNA-mRNA Network in the Kidneys of Wilson’s Disease Mice

**FIGURE 4:**
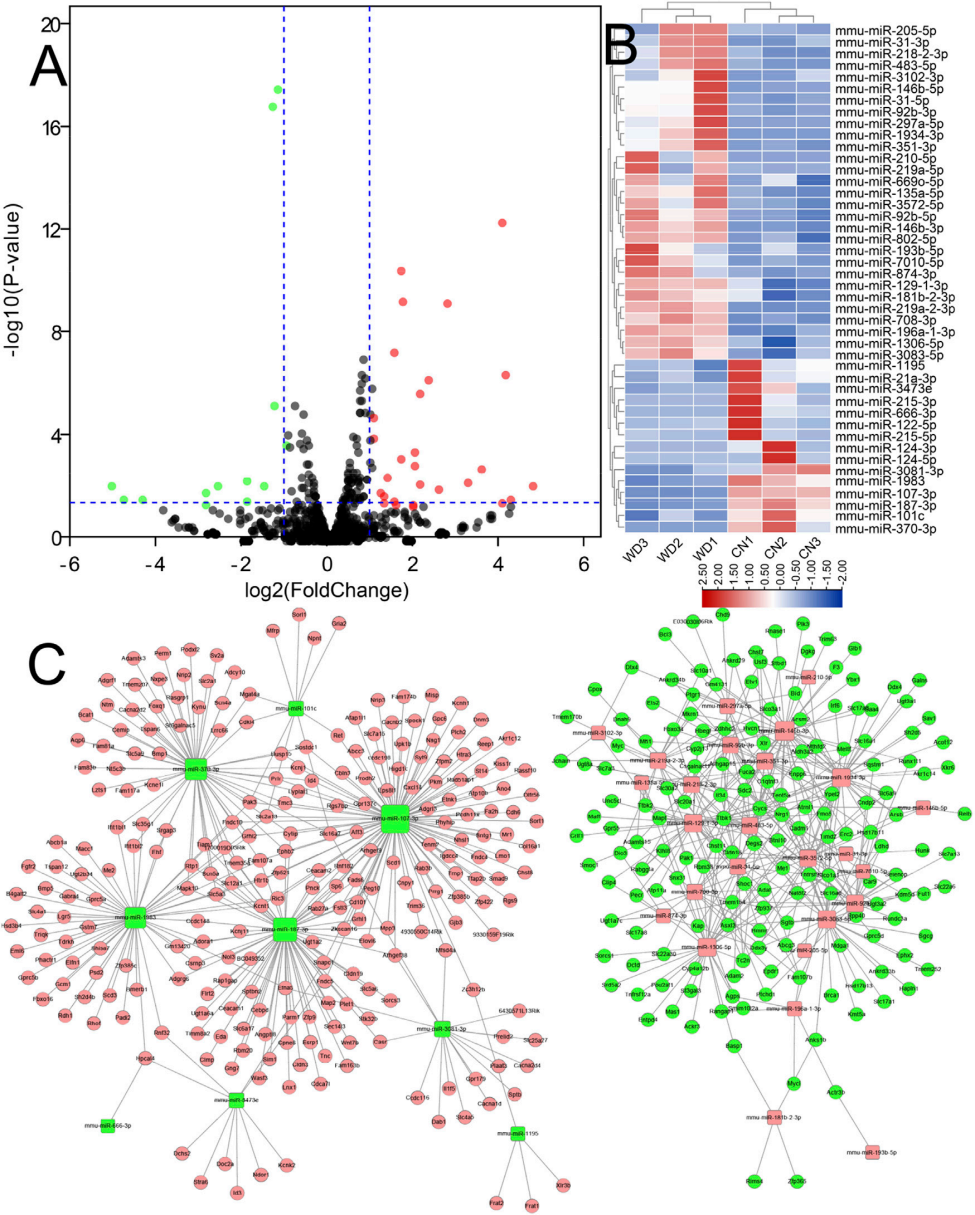
DEmiRs between WD and control groups. **(A)** DEmiRs displayed on a volcano plot. Blue and red indicate > two folds of decrease and increase in expression, respectively (*p* < 0.05). Gray indicates no significant difference. **(B)** DEmiRs displayed *via* hierarchical clustering; numbers indicate the samples used for sRNA-seq. **(C)** The miRNA-mRNA network constructed using DEmiRs and DEPs that were identified using sRNA-seq and RNA-seq. Pink nodes indicate upregulated RNAs and green nodes downregulated RNAs. Square nodes represent miRNAs. Circular nodes represent protein-coding genes.

The expression of miRNAs in the WD and CN kidneys was identified using sRNA-seq. In total, we identified 1,068 miRNAs from six samples. Further, compared to the CN group, 45 DEmiRs were screened in WD kidneys, including 30 upregulated and 15 downregulated (*p* < 0.05, log_2_ |fold change| > 1) ones. Volcano and hierarchical clustering plots of DEmiRs are shown in Figures 4A,B, respectively.

The targets of DEmiRs were predicted, and the correlation between DEMs and DEmiRs were identified. The miRNA-mRNA network was constructed (Figure 4C), which consisted of 35 DEmiRs, 416 DEMs, and 718 interactions between DEmiRs and DEMs.

### Identification of DEcircRs and Construction of the circRNA-miRNA Network in Kidneys of Wilson’s Disease Mice

**FIGURE 5:**
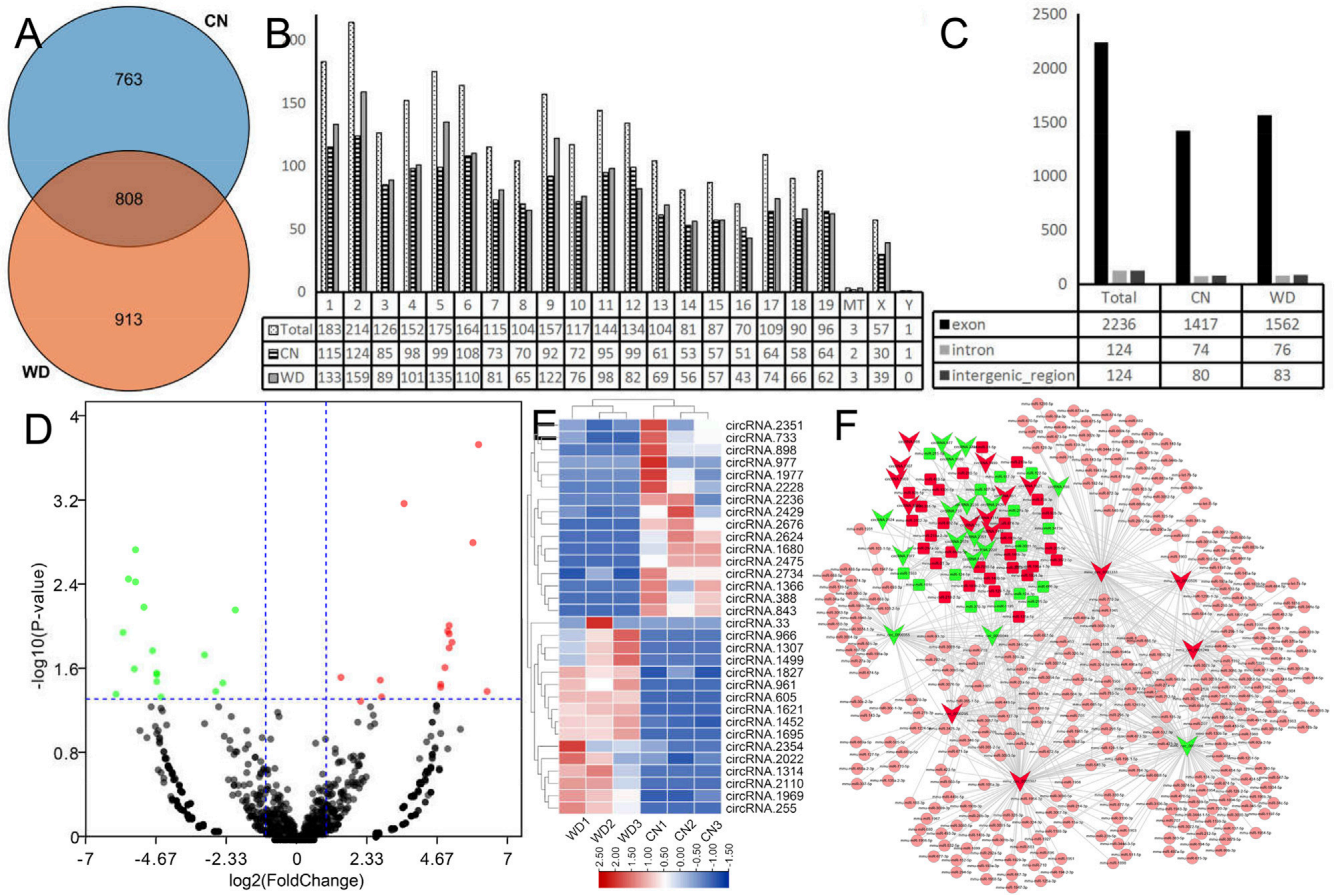
Landscape of circRNAs in WD mouse kidneys. **(A)** The Venn plot of circRNAs in the WD and CN groups. **(B)** Bar diagram showing the distribution of circRNAs in mouse chromosomes. **(C)** Bar diagram showing the different categories of circRNAs. **(D)** DecircRs displayed on a volcano plot. Blue and red indicate >two folds of decrease and increase in expression, respectively (*p* < 0.05). Gray indicates no significant difference. **(E)** DEcircRs displayed *via* hierarchical clustering; numbers indicate the samples used for RNA-seq. **(F)** DEcircRs-miRNA network constructed using DEcircRs and DEmiRs that were identified using RNA-seq and sRNA-seq. Red nodes indicate upregulated RNAs and green nodes downregulated RNAs. Square nodes represent miRNAs. V-shaped nodes represent circRNAs. Pink nodes indicate no significant difference in miRNA expression.

We characterized circRNA expression *via* deep RNA-seq for three CN and three WD kidney samples. In total, we identified 2,484 circRNAs from six samples, and 808 circRNAs were detected in both WD and control groups (Figure 5A). We analyzed the distribution of the identified circRNAs on the mouse chromosomes (Figure 5B). The results showed that all chromosomes generated circRNAs, although chrY only produced one circRNA, and three circRNAs were generated from mitochondria. Furthermore, we analyzed the identified circRNAs and found that most of them were transcribed from the protein-coding exons, some from introns and intergenic circRNAregions (Figure 5C).

DEcircRs in the WD and CN groups were successfully identified using EdgeR analysis and visualized *via* heatmap and volcano plots (Figures 5D,E). Finally, 32 DEcircRs were identified, including 16 upregulated and 16 downregulated circRNAs.

DEcircR-miRNA targets were predicted using the custom-made software. All DEcircRs, excluding circRNA.2354, possessed miRNA binding sites, circRNA.2110 (mmu_circ_0001333), circRNA. 2022 (mmu_circ_0001387), and circRNA.2475 (mmu_circ_0001566) interacted with more than one hundred miRNAs, and the DEcircRs-miRNA network was constructed with 31 DEcircRs and 348 miRNAs (Figure 5F).

### Construction of the DEcircR-DEmis-DEM Regulatory Network

**FIGURE 6:**
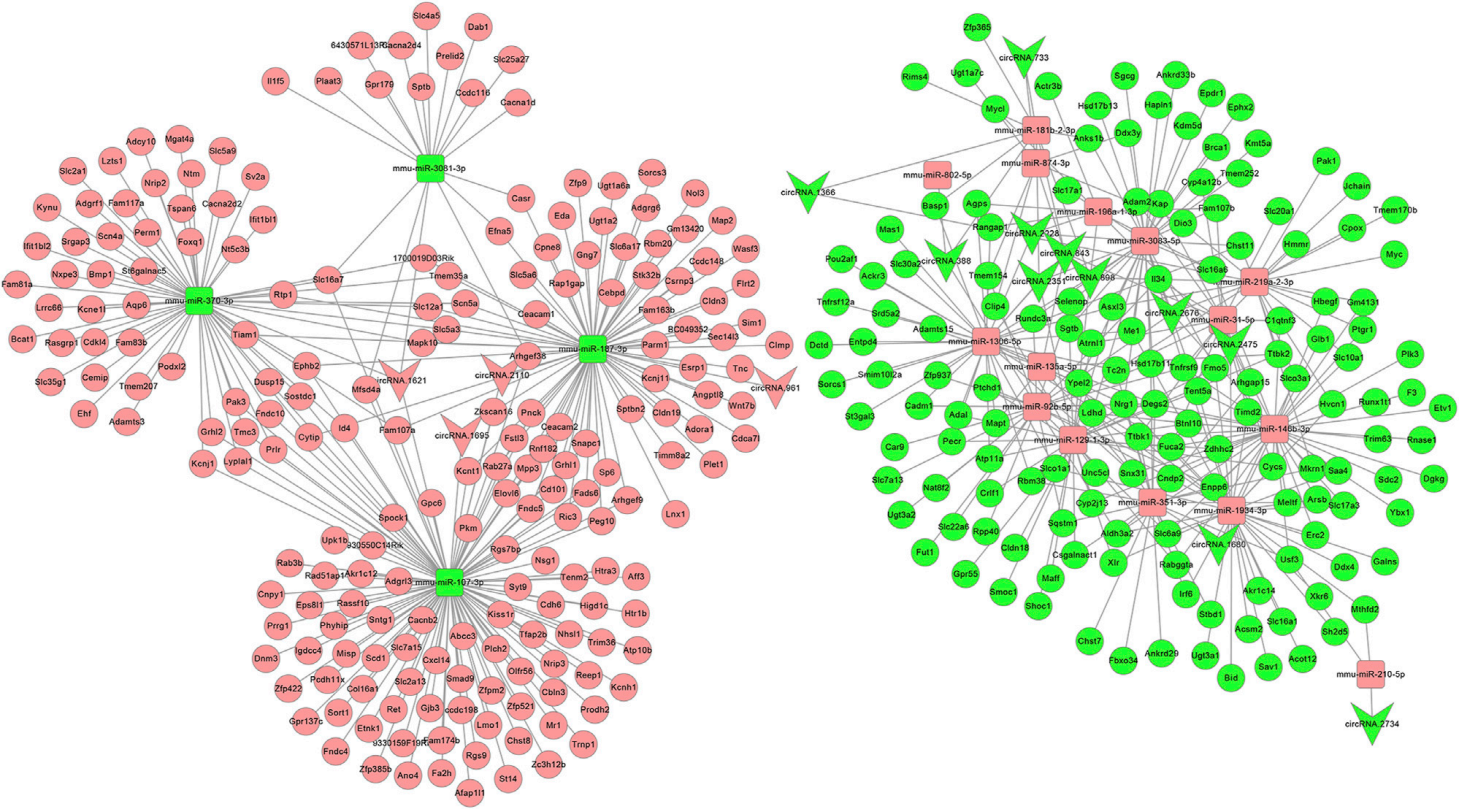
The circRNA ceRNA network. V-shaped, square, and circle nodes represent circRNA, miRNA, and mRNA, respectively. Red and green represent upregulation and downregulation, respectively.

After the DEcircRs-miRNA network was constructed, we selected the DEcircR-DEmiR pairs with a negative correlation and merged them with the miRNA-mRNA network to construct a ceRNA network. Finally, we constructed a ceRNA network consisting of 15 DEcircRNAs (four upregulated and 11 downregulated), 18 DEmiRNAs (14 upregulated and four downregulated), and 352 DEmRNAs (205 upregulated and 147 downregulated). Cytoscape 3.2.8 was employed to draw the ceRNA network (Figure 6).

### Functional Enrichment Analysis and Protein-Protein Interaction Analysis of the ceRNA Network

**FIGURE 7:**
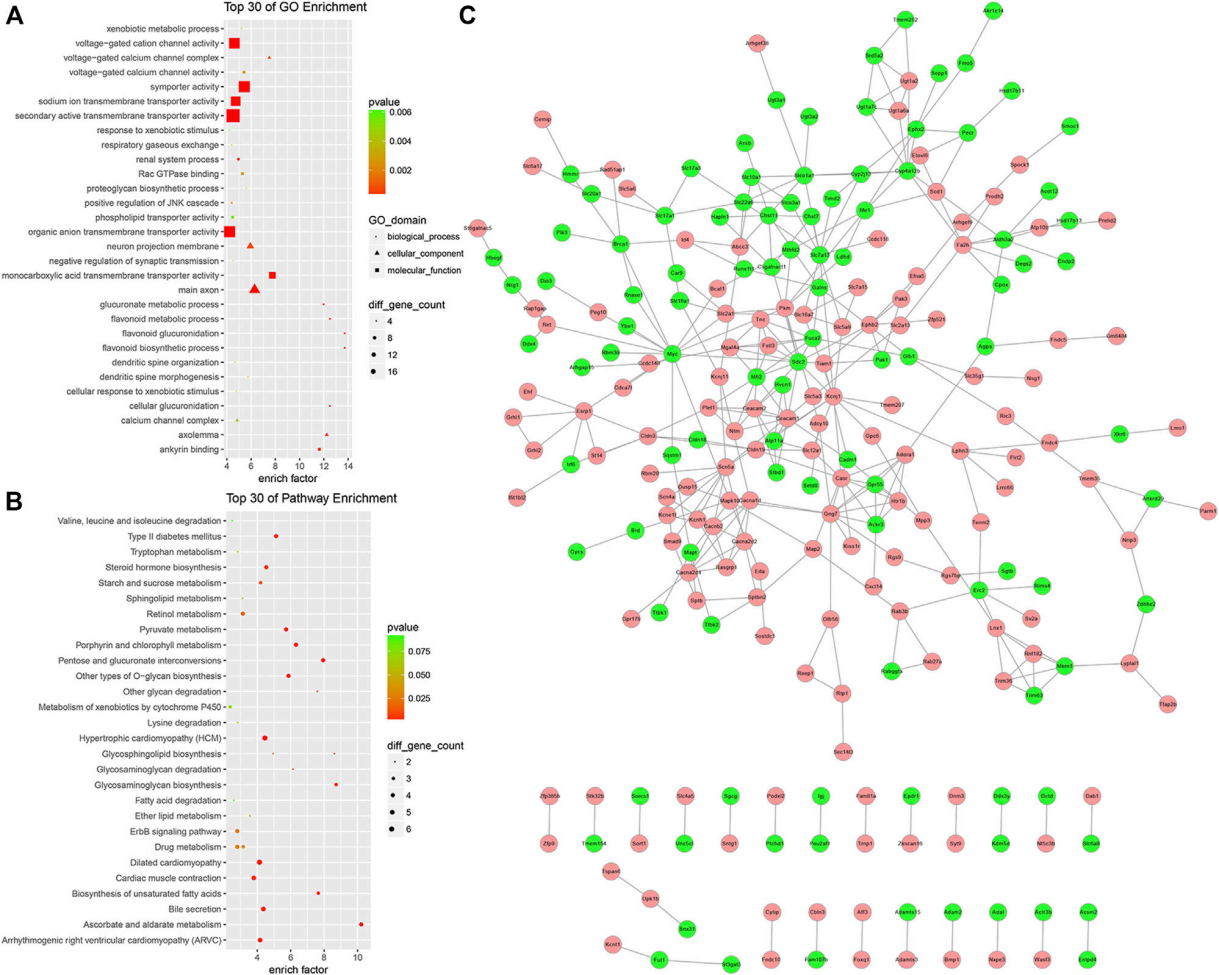
GO enrichment analysis, KEGG enrichment analysis, and PPI network of the circRNA ceRNA network. **(A)** GO Enrichment annotation of circRNAs. **(B)** KEGG pathway enrichment of circRNAs. **(C)** PPI network of the circRNA ceRNA network. Pink and green nodes denote upregulated and downregulated protein-coding genes, respectively. PPI, protein-protein interaction.

To further understand the function of the circRNA-related ceRNA regulatory network. GO and KEGG pathway enrichment analyses were performed, identifying 352 protein-coding mRNAs. The top 30 GO and KEGG pathway terms are presented in Figures 7A,B. The ceRNA network was significantly enriched in the BP terms anion transport, ion transport, flavonoid metabolic process, transmembrane transport, sodium ion transport, monovalent inorganic cation transport, cation transport, metal ion transport; the CC terms the intrinsic component of plasma membrane, integral component of plasma membrane, plasma membrane, cell periphery, main axon, intrinsic component of membrane, integral component of membrane, axolemma, synapse, and cation channel complex; the MF terms transmembrane transporter activity including the secondary active, anion, ion, substrate-specific, ion, and voltage-gated ion channel. In addition, the significantly enriched KEGG pathway terms were metabolism (ascorbate and aldarate, pentose and glucuronate interconversions, glycosaminoglycan, porphyrin and chlorophyll, unsaturated fatty acids, and pyruvate), hypertrophic cardiomyopathy (HCM), ErbB signaling pathway, ECM-receptor interaction, and MAPK signaling pathway. As shown in Figure 7C, we found dozens of hub genes in the PPI network, and these included *Myc*, *Gng7*, *Sdc2*, *Scn5a*, *Slc7a13*, *Casr*, *Ceacam1*, *Kcnj1*, *Mfi2*, *Ceacam2*, *Cyp4a12b*, *Fa2h*, and *Slco1a1*.

### qRT-PCR Validation of Differentially Expressed Genes in the ceRNA Network

**FIGURE 8:**
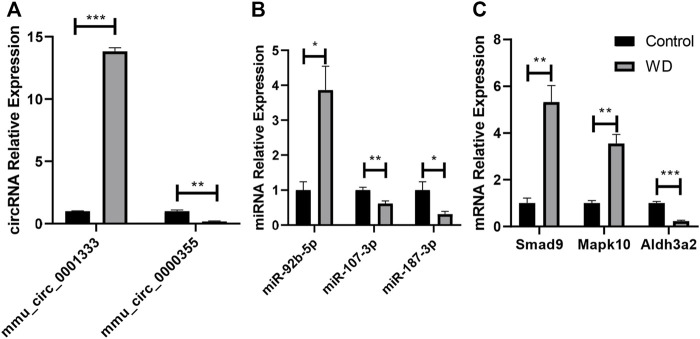
Validation of DE RNAs selected. **(A)** circRNAs; **(B)** miRNAs; **(C)** protein-coding genes. Expression was normalized to U6 for miRNAs and GAPDH for circRNAs and mRNAs as internal controls. Data are presented as the mean ± standard deviation (*n* = 3, *p < 0.05, **p < 0.01, ***p < 0.001).

As shown in Figure 8, it was confirmed that the expression of two circRNAs (including one upregulated circRNA, mmu_circ_0001333; one downregulated circRNA, mmu_circ_0000355; Figure 8A), three miRNAs (including one upregulated miRNA, miR-92b-5p; two downregulated miRNAs, miR-107-3p and miR-187-3p; Figure 8B), and three mRNAs (including two upregulated mRNAs, *Smad9* and *Mapk10*; one downregulated mRNA, *Aldh3a2*; Figure 8C) aligned with the RNA-seq results, indicating that our study was stable and repeatable.

## Discussion

Most studies focus on liver pathology and neuropathology in WD. In this study, we systematically elucidated the changes in mRNA, miRNA, and circRNA expressions in renal tissues, and constructed a circRNA-related ceRNA network of WD.

To the best of our knowledge, this is the first study to explore circRNA, miRNA, and protein-coding gene expression profiling in WD by performing next-generation RNA-seq. We discovered several DEmRs, DEmiRs, and DEcircRs, as well as their related pathways. Furthermore, we performed primary validation of the identified RNAs. Our results provide a new understanding of the underlying mechanism of WD pathogenesis.

We detected the expression profiles of circRNAs, miRNAs, and protein-coding genes, and identified 32, 45, and 1,623 differentially expressed circRNAs, miRNAs, and protein-coding genes, respectively, that may include potential regulators and explained, at least in part, the pathological mechanism of renal injury associated with WD.

Renal fibrosis is the ultimate common pathology of many chronic and progressive kidney diseases, and the typical characteristics include increased extracellular matrix (ECM) production, decreased ECM degradation, imbalance of cell-matrix interactions, infiltration of inflammatory cells, and transformation of the resident cells. Transforming growth factor-β (TGF-β) is a dimeric peptide that can play a multi-functional role in cell proliferation, differentiation and immune response. One of the principal biological effects is to regulate ECM accumulation (Border and Noble, 1994). TGF-β can induce the upregulation of ECM components, including adhesive proteins, collagens, and proteoglycans (Okuda et al., 1990). The conditioned medium of cultured glomerulus induces an increase in the synthesis of proteoglycan in normal mesangial cells. This response mimics the effect of the exogenous transforming growth factor-β, and it can be blocked by TGF-β. TGF-β can also inhibit the synthesis of metalloproteinases and induce that of metalloproteinase inhibitors, leading to the inhibition of matrix degradation (Tomooka et al., 1992). Glomerular manifestations of nephritis include the inhibition of plasminogen activator (PA) and promotion of plasminogen activator inhibitor-1 (PAI-1). Adding TGF-β to normal glomeruli can significantly reduce the activity of PA and increase the synthesis of PAI-1 (Tomooka et al., 1992). TGF-β changes the expression of integrins and regulates their relative proportions on the cell surface, which can promote adhesion to the matrix (Kagami et al., 1993). In the glomerulus, the mesangial expression of α1β1 and α5β1 integrins and their ligands (such as laminin, collagen, and fibronectin) is directly proportional to that of TGF-β1. TGF-β also induces the transformation of resident cells (Wu et al., 2013). In the obstructive renal fibrosis model, TGF-β activates the pericyte-myofibroblast transformation of renal tubular epithelial cells, and this phenotypic change is affected by anti-TGF-β1 antibody or TGF-β type I inhibitor (Wu et al., 2013).

Here, we found that 61 DEPs were enriched in the extracellular matrix (GO: 0031012, *p* = 8.61 × 10^−7^), and some classic signaling pathways were also significantly enriched, such as ECM-receptor interaction (mmu04512, 14 DEPs, *p* = 2.57 × 10^−3^) and TGF-β signaling pathway (mmu04350, 13 DEPs, *p* = 8.16 × 10^−3^). The circRNA ceRNA network showed that *Smad9* could be regulated by the mmu_circ_0001333/miR-107-3p axis, which was also confirmed using qRT-PCR.

WD is characterized by abnormal copper transport caused by *Atp7b* mutation. We also identified dozens of significantly enriched transport GO terms, such as anion transport, ion transport, transmembrane transport, lipid transporter activity, lipid transport, xenobiotic transport, and metal ion transport. Notably, nearly one hundred metabolism-related GO terms were also significantly enriched, including glucuronate, flavonoid, monocarboxylic acid, xenobiotic, steroid, organic acid, carboxylic acid, lipid, and collagen. PPAR signaling pathway, which is involved in the metabolism of FFAs, lactate, 3-hydroxybutyrate, citrate, pyruvate, α-ketoglutarate, glycerol, proline, lipid, glucose, and other amino acids. Specifically, we found 16 abnormally expressed genes in WD. Previous evidence shows that the PPAR signaling pathway participates in different kidney diseases (Corrales et al., 2018). Our results showed that the changes in the PPAR signaling pathway might lead to kidney injury in WD.

Limitations of this study included the small number of mice for each group, which should be increased in follow-up experiments to verify our findings. In addition, more experiments are needed to verify the results of this study. For example, mesalazine might play a therapeutic role in WD by regulating the TGF-beta signaling pathway. Therefore, it is necessary to study the effects of mesalazine on the TGF-β signaling pathway further, to improve our understanding of the therapeutic effect of mesalazine in WD.

## Conclusion

In summary, we screened the DE mRNAs, circRNAs, and miRNAs in the kidneys of TX-J mice with WD, and constructed the circRNA-miRNA-mRNA network. Our study is the first to evaluate the circRNA, miRNA, and mRNA profiles, as well as the ceRNA network in the kidney samples of WD mice, and also the first to reveal that this specific ceRNA network might participate in the pathogenesis of kidney injury in WD. The construction of the ceRNA network could help further understand the interaction between circRNAs, miRNAs, and protein-coding genes, and provide us with new insights into the underlying molecular mechanisms. This new ceRNA network will assist a better understanding of kidney injury in WD, and help identify therapeutic targets.

## Data Availability

The datasets presented in this study can be found in online repositories. The names of the repository/repositories and accession number(s) can be found below: https://bigd.big.ac.cn/bioproject/browse/ PRJCA007128, PRJCA007128.
